# Supplementary material for: Malaria and dengue in Hodeidah city, Yemen: High proportion of febrile outpatients with dengue or malaria, but low proportion co-infected
Source: PLoS One. 2021 Jun 25;16(6):e0253556. doi: 10.1371/journal.pone.0253556 (PMC8232408; doi:10.1371/journal.pone.0253556)
Supplement: S1 Table — (DOCX) [file pone.0253556.s002.docx]

**Questionnaire** (English version)

**Project title:** Assessment of the burden of concurrent infections with malaria and dengue among febrile patients in Hodeidah city, Yemen

- District: ____________Hospital:_____________ Date of survey: ____________________
- Patient’s name: _____________________________District code: ____ Patient’s ID: ____

1. **Sociodemographic data**

**Gender** (1 male**;** 2 female): ____ **Age** (years/months): ____ ____

**Education status** (0 no formal education**;** 1 primary**;** 2 secondary**;** 3 University and above): ____

**Employment status** (0 unemployed**;** 1 public service employee**;** 2 private sector employee:____ **Other** (specify):____________________ **Household size** (members)**:**  ____

1. **Clinical data**

| **Temperature** (℃): ____ |  |
| --- | --- |
| **Sweating** (0 no**;** 1 yes): ____ | **Chills** (0 no**;** 1 yes): ____ |
| **Headache** (0 no**;** 1 yes): ____ | **Muscle pain** (0 no**;** 1 yes): ____ |
| **Joint pain** (0 no**;** 1 yes): ____ | **Presence of skin rash** (0 no**;** 1 yes): ____ |
| **Retro-orbital/ ocular pain** (0 no**;** 1 yes): ____ | **Vomiting** (0 no**;** 1 yes): ____ |

1. **Laboratory data** (To be filled after laboratory investigations)
2. **Malaria microscopy**

| **Result** (0 negative**;** 1 positive): ____ | | | |
| --- | --- | --- | --- |
| **If positive:** | | | |
| ***Plasmodium* species:** | *P. falciparum* ____ | *P. vivax* ____ | **Others** (specify):___________ |

1. **Malaria Combo RDT**

| **Result** (0 negative**;** 1 *P. falciparum* only**;** 2 Non-falciparum species**;** 3 Falciparum and non-falciparum species): ____ |
| --- |

1. **Dengue RDT**

| **Result** (0 negative**;** 1 IgM-positive**;** 2 IgG-positive**;** 3 NS1-positive**;** 4 IgM- and IgG-positive**;** 5 NS1 and IgM- and/or IgG-positive ): ____ |
| --- |

Signature of data collector

-------------------------------------

**Questionnaire** (Arabic version)

**اسم المشروع**: تقييم عبء العدوى المتزامنة بالملاريا وحمى الضنك بين مرضى الحمى في مدينة الحديدة، اليمن

- المديرية:________________ المستشفى:___________ تاريخ المسح:________________
- اسم المريض:________________________كود المديرية: ____ رقم المريض: ____

**أ. البيانات الاجتماعية الديموغرافية**

- **الجنس** (1 ذكر؛ 2 أنثى): ____ **العمر** (سنة/ شهر) ____ ____
- **الوضع التعليمي** (0 بدون تعليم رسمي**؛** 1 ابتدائي**؛** 2 ثانوي**؛** 3 جامعي وما فوق): ____
- **الوضع الوظيفي** (0 عاطل عن العمل**؛** 1 موظف خدمة عامة**؛** 2 موظف قطاع خاص): ____ أخرى (حدّد):_____________________
- **عدد أفراد المنزل:** ____

**ب. البيانات السريرية**

|  | **درجة الحرارة** (℃): ____ |
| --- | --- |
| **قشعريرة** (0 لا**؛** 1 نعم):____ | **تعرق** (0 لا**؛** 1 نعم):____ |
| **ألم في العضلات** (0 لا**؛** 1 نعم):____ | **صداع** (0 لا**؛** 1 نعم):____ |
| **طفح جلدي** (0 لا**؛** 1 نعم):____ | **ألم في المفاصل** (0 لا**؛** 1 نعم):____ |
| **تقيؤ** (0 لا**؛** 1 نعم):____ | **ألم في حجاج العين/العين** (0 لا**؛** 1 نعم):____ |

**جـ. البيانات المعملية**

1. **الفحص المجهري للملاريا** (0 سلبي؛ 1 إيجابي): ____

إذا كانت النتيجة إيجابية، فإن نوع طفيل الملاريا:

| *P. falciparum* ____ | *P. vivax* ____ | **أخرى** (حدد):___________________ |
| --- | --- | --- |

1. **نتيجة الفحص السريع للملاريا** (0، سلبي**؛** 1*P. falciparum*  **؛** 2Falciparum and non-falciparum species **؛** 3Non-falciparum species ): ____
2. **نتيجة الفحص السريع لحمى الضنك** (0 سلبي**؛** 1IgM-positive **؛** 2IgG-positive **؛** 3 NS1-positive **؛** 4 IgM- and IgG-positive **؛** 5 NS1 and IgM- and/or IgG-positive) : ____

**توقيع جامع البيانات**

---------------------------
