# Supplementary material for: Malaria and dengue in Hodeidah city, Yemen: High proportion of febrile outpatients with dengue or malaria, but low proportion co-infected
Source: PLoS One. 2021 Jun 25;16(6):e0253556. doi: 10.1371/journal.pone.0253556 (PMC8232408; doi:10.1371/journal.pone.0253556)
Supplement: Attachment — Submitted filename: Plos reviewer.docx [file pone.0253556.s005.docx]

Reviewer attachments

Author has addressed all queries. The article may be accepted
