# Supplementary material for: Malaria and dengue in Hodeidah city, Yemen: High proportion of febrile outpatients with dengue or malaria, but low proportion co-infected
Source: PLoS One. 2021 Jun 25;16(6):e0253556. doi: 10.1371/journal.pone.0253556 (PMC8232408; doi:10.1371/journal.pone.0253556)
Supplement: Attachment — Submitted filename: Response to Reviewers.docx [file pone.0253556.s006.docx]

Response to Reviewers’ Comments

**Dear Editor,**

Thank you for giving us this second opportunity to further improve the quality of our manuscript. Our thanks are also due to the Reviewers for their helpful comments and concerns. The manuscript has been revised after considering these comments and carefully adhering to the editorial requirements of PLOS ONE. Changes in the revised manuscript are made with track changes. The line numbers mentioned below refer to those in the revised manuscript with track changes. Detailed responses to the comments raised are as follows:

**Responses to the comments by the Academic Editor:**

**Comment**: “*Thank you for submitting your manuscript to PLoS ONE. After careful consideration, we feel that your manuscript will likely be suitable for publication if the authors revise it to address critical points raised now by the reviewer. According to reviewer, there are some specific areas where further improvements would be of substantial benefit to the readers. For your guidance, a copy of the reviewers' comments was included below”*

**Response**: We thank the Academic Editor and the Reviewers for the helpful comments and careful consideration to improve the quality of our manuscript. We addressed the additional concerns and issues raised by Reviewer 2 in the revised version of the manuscript. Because World Malaria Report is issued annually to provide statistics on malaria during the previous year, we updated the figures on malaria epidemiology in the revised manuscript as per the latest edition of the report issued in 2020 instead of those provided in the 2019 edition (Lines 57& 58 and Lines 69 & 70 of the revised manuscript) and updated citation #1 in the list of references, accordingly. In addition, we proofread the manuscript for language issues and fixed the minor issues we found (marked with track changes in the revised manuscript).

**Responses to PLOS ONE requirements:**

**Comment**: “*Please review your reference list to ensure that it is complete and correct. If you have cited papers that have been retracted, please include the rationale for doing so in the manuscript text, or remove these references and replace them with relevant current references. Any changes to the reference list should be mentioned in the rebuttal letter that accompanies your revised manuscript. If you need to cite a retracted article, indicate the article’s retracted status in the References list and also include a citation and full reference for the retraction notice.”*

**Response**: We double-checked the reference list of our manuscript, which was originally created using EndNote software, for its completeness, correctness and formatting according to PLOS ONE’s style after removing the EndNote links. We did not cite retracted papers in our manuscript. We added one citation to our revised manuscript (#66; Prasad et al.) to enrich the discussion added in response to the comment by Reviewer 2.

**Responses to the comment by Reviewer 1:**

**Comment**: “*The authors have addressed all the queries as brought about in the review. The article may be accepted.”*

**Response**: We thank the Reviewer for the supportive feedback. We just noted that the Reviewer answered with “No” to question #4 “*Have the authors made all data underlying the findings in their manuscript fully available?*”, but we confirm that we made all data fully available and uploaded an Excel data file with the first version of our revised manuscript submitted to the journal.

**Responses to the comment by Reviewer 2:**

**Comment**: “*Authors responded to most comments and incorporated most of the suggestions. While the major issue with underestimation of infection frequencies still exists, in most settings, RDT and microscopy are the methods used for evaluation of malaria and dengue co-infections. A short discussion on other potential co-infections that may be present but have not been investigated in the current study, may be included.*”

**Response**: We thank the Reviewer for this important comment. Following the suggestion above, we added a discussion paragraph on other potential co-infections that may be present but have not been investigated in the present study (Lines 350–369 of the revised manuscript). We also added a sentence about this important issue to the recommendations of the study (Lines 396–398) to highlight its importance.

Additionally, we elaborated in the first version of our revised manuscript on the underestimation of mono- and co-infection with either type of infection based on the diagnosis of malaria with microscopy and RDTs and dengue with RDTs in light of the Reviewer’s comments on our original submission (lines 244–272: “Malaria diagnosis in the present study using microscopy and antigen detection using RDTs may underestimate….. preferably as a single format, for both types of infection has been suggested [14].”

We hope that we addressed the comments raised by the Editor and Reviewers that contributed to further improving the quality of our manuscript. We also hope that our revised manuscript is accepted for publication in **PLOS ONE**, and we are pleased to receive any further comments or suggestions.

Best regards,

Rashad Abdul-Ghani, PhD

The Corresponding Author
